# Case Report: Semantic Variant Primary Progressive Aphasia With Impaired Verbal Word Discrimination

**DOI:** 10.3389/fneur.2022.873735

**Published:** 2022-06-16

**Authors:** Nobuko Kawakami, Ayumi Morita, Shigenori Kanno, Nanayo Ogawa, Kazuo Kakinuma, Yumiko Saito, Erena Kobayashi, Wataru Narita, Kyoko Suzuki

**Affiliations:** ^1^Department of Behavioral Neurology and Cognitive Neuroscience, Tohoku University Graduate School of Medicine, Sendai, Japan; ^2^Department of Rehabilitation, Tohoku University Hospital, Sendai, Japan; ^3^Department of Neurology and Stroke Medicine, Yokohama City University, Yokohama, Japan

**Keywords:** semantic variant, primary progressive aphasia, verbal word recognition, verbal word discrimination, word discrimination, auditory agnosia, nonverbal sound discrimination

## Abstract

Some patients with primary progressive aphasia (PPA) present with various types of hearing deficits. Research on the auditory function and speech sounds in PPA, including temporal, phonemic, and prosodic processing, revealed impairment in some of these auditory processes. Many patients with PPA who present with impaired word recognition subsequently developed non-fluent variant PPA. Herein, we present a patient with semantic variant PPA (svPPA) who demonstrated impaired verbal word discrimination. Audiological examinations revealed normal auditory brainstem responses and slightly impaired pure-tone perception. By contrast, verbal word discrimination and monosyllable identification were impaired, and temporal auditory acuity deteriorated. Analyses of brain magnetic resonance images revealed a significant decrease in the gray matter volume in bilateral superior temporal areas, predominantly on the left, compared with those of patients with typical svPPA, which appeared to be associated with impaired word recognition in our patient.

## Introduction

Primary progressive aphasia (PPA) is a clinical syndrome characterized by progressive loss of language function and is associated with various neurodegenerative diseases. Based on its diagnostic criteria, PPA is classified into three variants: non-fluent/agrammatic variant PPA (nfvPPA), semantic variant PPA (svPPA), and logopenic variant PPA (lvPPA) (1). However, several patients do not meet the diagnostic criteria for PPA, which suggests the existence of atypical PPA (2–5). The previous studies have reported that some patients with PPA present with impaired recognition of auditory stimuli, including progressive word deafness (6–10), auditory agnosia in its broader sense (6–8, 11, 12), and pure auditory agnosia restricted to non-verbal sounds (13).

The neural bases of verbal and non-verbal sound processing in the patients with PPA were explored through neuroradiological examinations. Studies of voxel-based morphometry (VBM) on brain magnetic resonance images (MRI) have revealed that impairment in auditory phonemic discrimination in lvPPA was positively correlated with the gray matter volume in the left angular gyrus (14), and the phonetic spectral processing deficit was associated with the gray matter volume in the left supramarginal gyrus (SMG) in nfvPPA and svPPA (15). Activation fMRI study examined activated brain areas during listening to sequences of spoken syllables with manipulation of temporal regularity, phonemic spectral structure, and pitch sequence entropy. Each variant of PPA exhibited a different distribution of abnormal activation in response to these auditory speech signal characteristics (16). Longitudinal fluorodeoxyglucose-positron emission tomography in a patient with PPA and environmental sound agnosia revealed increased hypometabolic areas in the left temporoparietal regions (13).

Some patients with svPPA demonstrated auditory agnosia for non-verbal sound (17–19), whereas impaired verbal word discrimination has not been reported in the patients with svPPA. Herein, we present a patient with svPPA who demonstrated impaired auditory word discrimination and recognition and temporal auditory acuity deficit. We performed neuroradiological studies to identify the brain regions associated with the auditory symptoms, which revealed marked atrophy in the perisylvian area, dominant on the left side, compared with our disease control patients with typical svPPA.

## Case Report

An 80-year-old, right-handed woman was referred to our hospital for a 3 year history of moderate amnesia and progressive difficulties in finding words and understanding conversations. She was a college graduate and had worked as a nurse until her mid-50 s. She was healthy except for bladder cancer surgery at the age of 60. At age 77, she demonstrated difficulty in finding words and diminished ability to recognize spoken words, which progressed in subsequent years. At age 78, in addition to the prominent aphasia, she demonstrated amnesia and slight behavioral change, such as compulsive washing of hands and dishes; however, they did not have a significant effect on her daily activities.

On admission to our hospital, she was alert and cooperative to the examination, but she was occasionally a little reluctant to, particularly memory tests. Sometimes, we had to repeat instructions because she could not understand the first time. However, she understood what should be done following repetitive instructions or written commands.

Frontal signs such as disinhibition were occasionally observed, but they were relatively mild. Neurological examination did not reveal motor or sensory disturbance and parkinsonian symptoms. She exhibited bilateral palmomental reflexes but no grasp reflex. Her autonomic functions were well preserved.

### Neuropsychological Assessment

Neuropsychological assessments were performed between the second and 18th days of hospitalization. Some of her performance seemed to be affected by her impaired listening comprehension or non-serious attitude. For her best performance, clear and plain instructions were repeated until she could understand them. The results of the neuropsychological tests are presented in Table 1.

**Table 1:** Performance on the neuropsychological tests.

**Neuropsychological tests**		**Results**	**Normal range**
**Mini-Mental State Examination (30)**		8	
**Digit span** (Forward, backward)		6, 3	
**Tapping span** (Forward, backward)		6, 2	
**Rey–Osterrieth Complex Figure Test**			
Copy (36)		36	
Delayed recall (36)		0	
Recognition (24)		18	
**Wechsler Memory Scale-Revised**			
Visual memory index		56	
**Wechsler Adult Intelligent Scale III**			
Performance IQ		76	
**Raven's Colored Matrices (37)**		21	
**Birmingham Object Recognition Battery: Object decision task**			
HARD A (32)		20	27.0 ± 2.2
EASY B (32)		6	30.5 ± 1.4
**Semantic Memory Task (16)**		10	16
**Western Aphasia Battery (Japanese version)**			
Aphasia quotient (100)		59.4	
Spontaneous speech	Information content (10)	5	
	Fluency (10)	8	
Auditory comprehension	Yes/No questions (60)	45	
	Auditory word recognition (60)	44	
	Sequential commands (80)	45	
Repetition (100)	Naming	91	
	Object naming (60)	0	
	Word fluency (20)	1	
	Sentence completion (10)	4	
	Responsive speech (10)	4	
Reading (10)		4.8	
Writing (10)		3.6	
Praxis (60)		50	
Drawing (30)		24	
Block design (9)		6	
Calculation (24)		14	
**Test of Lexical Processing in Aphasia**			
Naming (200)		31	191.80
Auditory comprehension (200)		137	199.50

*Semantic Memory Task; The original task in which a participant is shown one target image and asked to choose the associated image from four others; similar to the Pyramid and Palm Trees test*.

Test scores on WAIS-III and Raven's Colored Progressive Matrices were nearly at the lower limit of normal performance. The Wechsler Memory Scale-Revised and Rey–Osterrieth Complex Figure Test indicated severely impaired visual memory. Apraxia, visual agnosia, and prosopagnosia were not observed.

Her speech was fluent without distortion or agrammatism. She demonstrated difficulty in finding words and visual confrontation naming. Although auditory comprehension of sentences and words was impaired, they were due to the difficulty in phonological word processing. Written word comprehension was much better than spoken word comprehension. Results of the Western Aphasia Battery Japanese version revealed anomia, poor spoken word comprehension, and poor writing, but speech production was relatively well preserved. The scores on the repetition task were good, but phonological errors in words were observed. She could read kana (phonogram) and regular kanji (morphogram) words, but she frequently made mistakes when reading irregular kanji words, indicating surface dyslexia (20). In addition, she demonstrated difficulty writing kanji but not kana. These features in Japanese suggested impairment of semantic memory for words (21) and were often observed in semantic dementia (22). On the standard tests in Japanese to examine naming and auditory comprehension of 100 high-frequency and 100 low-frequency words, she scored 31/200 on the naming task and 137/200 on the auditory comprehension task. We examined written comprehension for 61 of the 63 words that could not be understood on the auditory comprehension task. We excluded two words because we selected the word that she presented a two-way anomia. Accordingly, her written word comprehension was correct for 31 words (50%).

To evaluate her non-verbal semantic memory, we used the object decision task of the Birmingham Object Recognition Battery and an original semantic memory task, similar to the Pyramid and Palm Trees test. We used the original semantic task because some items in the Pyramid and Palm Trees test are unfamiliar to Japanese people. The results of these tests revealed that the patient also had non-verbal semantic memory impairment. Thus, the patient experienced prominent language deficits throughout the clinical course with verbal and non-verbal semantic memory impairment, which fulfilled the diagnostic criteria for svPPA (1).

By contrast, the patient demonstrated difficulty in repeating and dictating spoken words, which is not a common feature in svPPA. Listening errors were improved by the repetition of target words, and the ability to repeat long sentences was relatively preserved. Therefore, we hypothesized that impaired detection of each phoneme may affect her ability to comprehend and repeat spoken words. The context could also help estimate the words in long sentences.

Accordingly, we performed a comprehensive examination of the higher auditory functions of the patient and conducted neuroradiological analyses to reveal the areas associated with the auditory symptoms.

### Elementary Auditory Functions

Auditory brainstem responses that were elicited with the standard protocol demonstrated normal waves. We used an adapted form of a standard clinical audiometry protocol in assessing frequencies of 500, 1,000, 2,000, and 4,000 Hz. The patient's pure-tone audiometry threshold was within the normal range for her age, with mean threshold values of 36.3 and 36.3 dB on the right and left, respectively.

### Higher Auditory Functions

To examine the higher auditory functions, we performed: (1) temporal auditory acuity and; (2) verbal sound discrimination and recognition. The results are presented in Table 2. Her ability to identify non-verbal sounds and nursery songs was also examined (Supplementary Table 1). We did not analyze these data in detail because several factors including word-finding impairment and semantic memory loss made further analyses difficult.

**Table 2:** Results of the examinations of auditory function.

	**Right**	**Left**	**Normal range**
**Pure-tone audiometry threshold (dB)**			
500 Hz	25	25	
1,000 Hz	35	35	
2,000 Hz	50	50	
4,000 Hz	45	35	
**Auditory brain responses (ms)**			
Wave I	1.58	2.00	<2.21
Wave II	3.73	3.79	<4.51
Wave V	5.69	5.93	<6.43
**Temporal auditory acuity**			
Click counting (counts/s)	5^*^	5^*^	9–11
Click fusion (ms)	8^*^	8^*^	1–3
Single mora recognition accuracy	75%^*^	80%^**^	80–100
2-mora word discrimination (36)	31^*^		35.82 (0.50)
2-mora non-word discrimination (36)	28^*^		35.14 (1.06)

* *Impaired performance relative to normal controls*;

** *lower limit of normal controls*.

#### Examinations of Temporal Auditory Acuity

Click counting and click fusion tests (23) were also performed. In the click counting test, the patient was asked to count the number of clicks per second. She was able to count five clicks per second in each ear, which was lower than the 9–11 clicks per second detected by normal controls (24). In the click fusion test, click sounds were delivered to each ear at various intervals between binaural pulses. The patient was requested to report whether one or two clicks were made. She exhibited binaural fusion at an interval of 8 ms, which was worse than the several milliseconds detected by normal controls (24).

#### Verbal Sound Recognition and Discrimination

**Figure 1:**
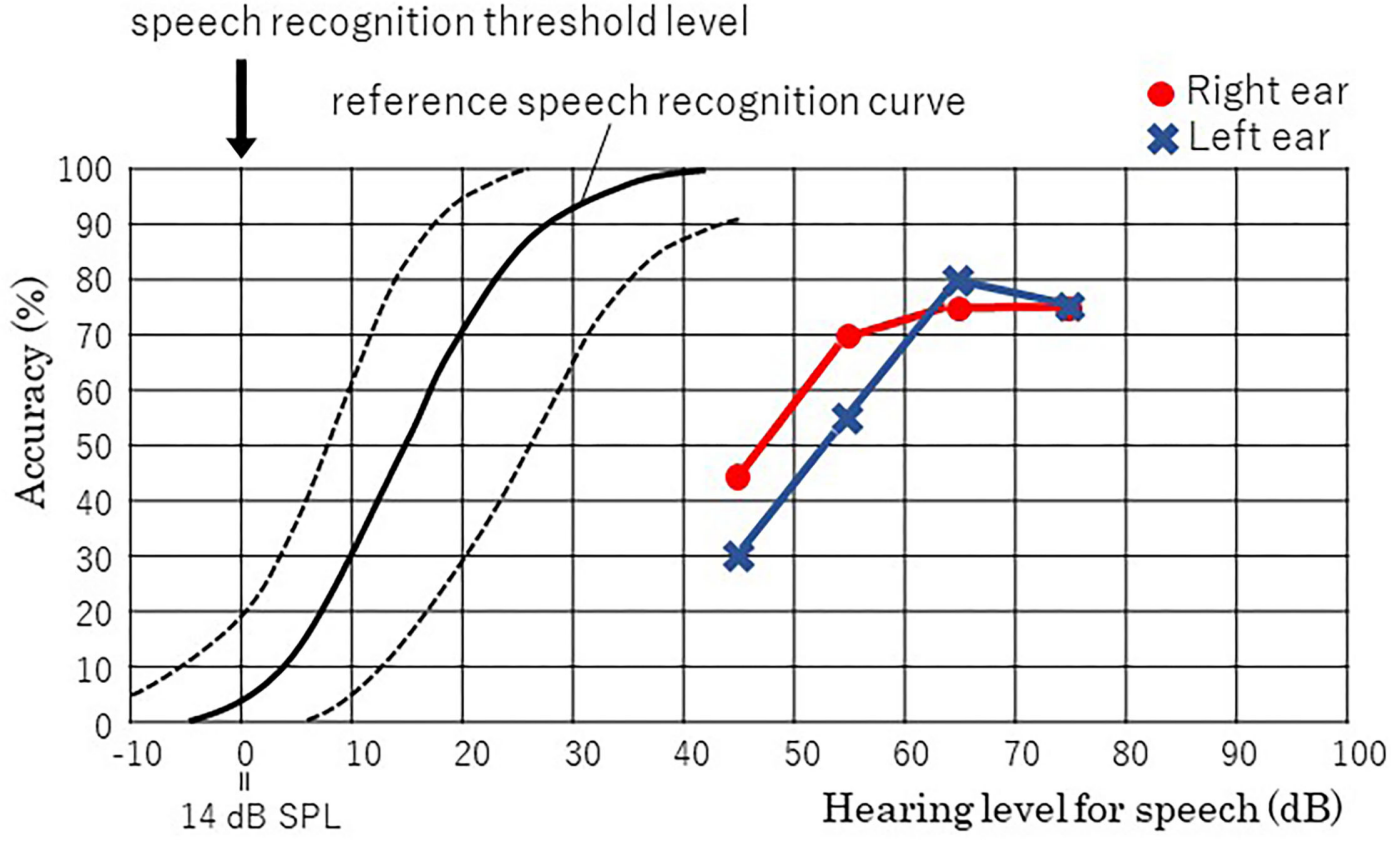
Results of the speech audiogram. The curve marked with a solid black line is the reference speech recognition curve and that between the curves marked with black dashed lines on both sides of it represent the normal range. The speech recognition scores of the right ear are marked by red circles and those of the left ear are marked by a blue cross, and those are connected by solid lines of the same color with marks. Both are out of the normal range, and the maximum recognition score in the phonetically balanced word list of the right ear is 75% at 65 dB and that of the left ear is 80% at 65 dB.

In Japanese, the ultimate minimum unit of a verbal phoneme sound is expressed as a “mora.” A mora was defined as one vowel or a unit of a consonant and a vowel, which corresponded to one kana letter. When measuring the speech recognition score in Japanese, the participants are requested to listen to a mora, a monosyllabic sound, produced verbally and to answer the corresponding kana letter by dictation (25). Because she was unable to write well enough, the test was performed by recitation. She recognizes 15/20 moras (75%) at 65 dB with the right ear and 16/20 moras (80%) at 65 dB with the left ear. The speech audiogram revealed significantly impaired speech recognition relative to the normal range advocated by the Japanese Audiological Society (25) (Figure 1). Her maximum recognition score in a phonetically balanced word list of the right ear was 75% (15/20 moras) at 65 dB and that of the left ear was 80% (16/20 moras) at 65 dB. Thus, a higher volume was needed to increase the accuracy, and speech recognition accuracy did not exceed 80% in either ear even at the suprathreshold level. The two-mora discrimination tasks using 36 words and 36 non-words were performed. She discriminated 31 of 36 words (normal range; 35.82 ± 0.50) and 28 of 36 non-words (normal range; 35.14 ± 1.06), which was worse than normal controls. These results support the hypothesis that the patient's ability to recognize phonemes was impaired, and she used the context of sentences to estimate the words.

### Neuroradiological Investigations

The MRI scans and ^123^I-iodoamphetamine single-photon emission computed tomography (^123^I-IMP-SPECT) were performed.

#### Magnetic Resonance Imaging

**Figure 2:**
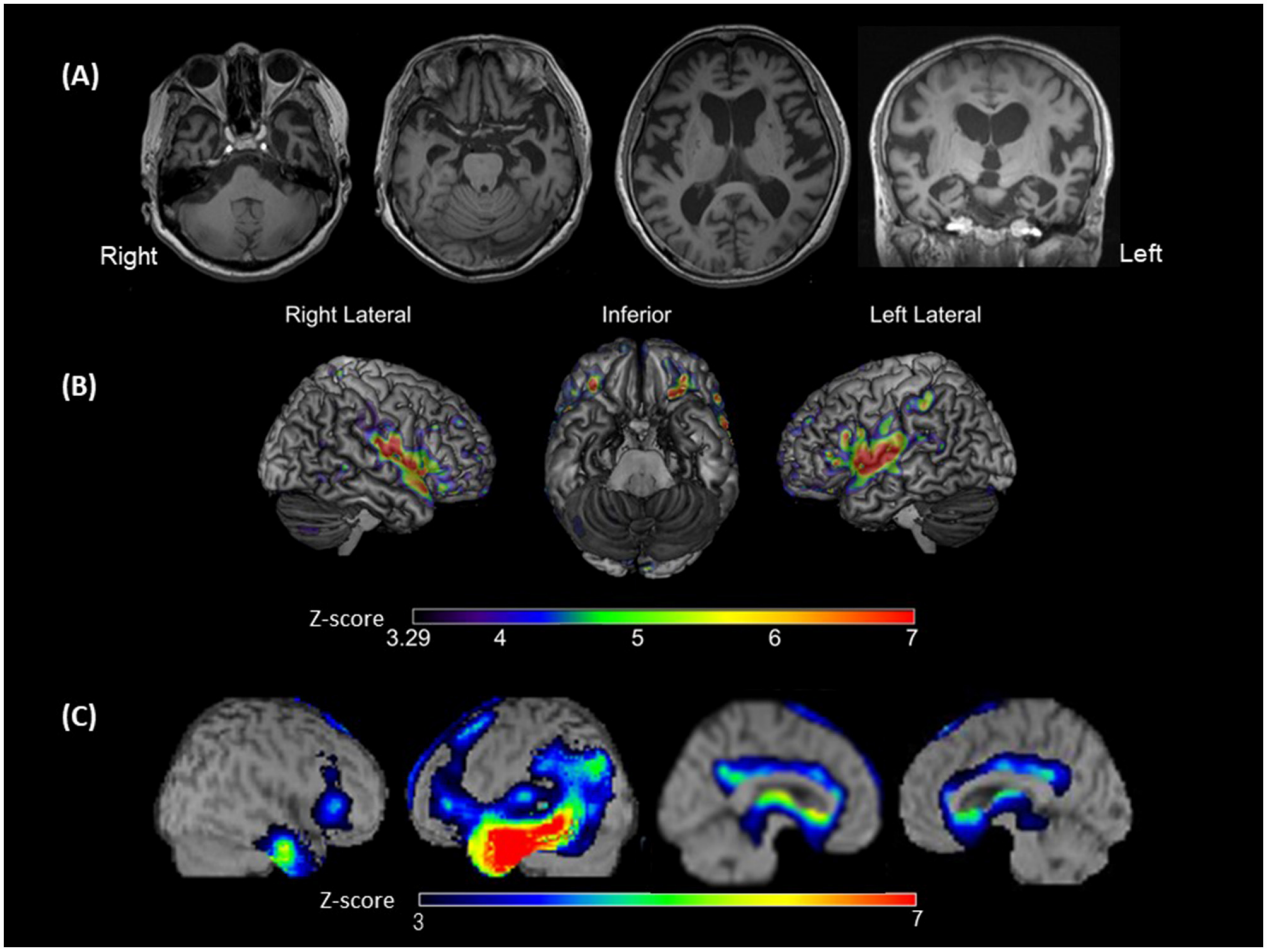
Brain magnetic resonance imaging (MRI), voxel-based morphometry on brain MRI (VBM), and single-photon emission computed tomography (SPECT). **(A)** Brain MRI showing left-sided predominant atrophy, especially in Heschl's gyrus, superior temporal gyri (STG), and the anterior and medial temporal areas; **(B)** VBM showing the significantly smaller gray matter volume areas in the bilateral STG, plana temporale, Rolandic area and frontal inferior opercula, and the left supramarginal gyrus of the patient compared with those observed in patients with typical svPPA (disease controls). The colored bars indicate the *Z*-values; **(C)** SPECT data analyzed with three-dimensional stereotactic surface projections showing predominant hypoperfusion of the temporal lobe, especially at the anterior temporal area and middle and inferior temporal gyrus. Hypoperfusion is expanded to the temporoparietal junction in the left hemisphere. The colored bars indicate the *Z*-values.

Eight days after admission, we performed an MRI of the brain using a 3-Tesla MAGNETOM Trio (Siemens Medical Solutions United States, Inc., PA, United States). Three-dimensional magnetization-prepared rapid acquisition with gradient echo (3D-MPRAGE) images demonstrated diffuse cerebral atrophy, which was especially marked in the anterior and medial parts of the left temporal lobe (Figure 2A).

##### Voxel-Based Morphometry

To identify brain regions of the patient in which gray matter volumes were smaller than those in patients with typical svPPA, we performed VBM using statistical parametric mapping software (SPM), version 12 (http://www.fil.ion.ucl.ac.uk/spm/). Three-dimensional spoiled gradient echo or 3D-MPRAGE image data of 10 patients with typical svPPA as disease controls (mean age, 64 ± 8.0 years; 7 women and 3 men) were used for the analysis. The imaging parameters used for the acquisition of structural imaging data are presented in Supplementary Table 2. The disease controls were retrospectively recruited from the database for the study of dementing illnesses from June 2009 to April 2021 in Tohoku University Hospital and were diagnosed by board-certified neurologists based on the PPA criteria (1).

The protocol of the study was approved by the Ethics Committee of Tohoku University Hospital (approval nos. 2006–19, 2014–1–278, 2018–1–024, 2019–1–156, and 2020–1–285).

A *Z*-score map of the present patient was created using the mean and standard deviation of the gray matter volume (normalized by the total intracranial volume) of each voxel in the disease controls and the following equation: *Z*-score = [(Control mean) – (Present patient's value)]/(control standard deviation). Significance was defined as a *Z*-score above 3.29 (α < 0.0005). The *Z*-score map is exhibited in Figure 2B. Compared with that of disease controls, in the present patient, gray matter volume was significantly smaller in the bilateral perisylvian areas, including the bilateral superior temporal gyri (STG), bilateral plana temporale (PT), bilateral Rolandic area and frontal inferior opercula, and left SMG.

##### Region of Interest-Based Analyses

We performed ROI-based analyses for gray matter volumes using the computational anatomy toolbox (CAT12, http://www.neuro.uni-jena.de/cat/) for SPM (26). The ROIs were defined based on the previous studies that have reported lesions that were associated with the manifestation of word deafness or auditory agnosia (12–16, 27–29). These ROIs were as follows: (i) left and right STG; (ii) left and right PT; (iii) left and right Heschl's gyri; (iv) left and right angular gyri; and (v) left and right SMG. A *Z*-score of each ROI was calculated by the same equation as that used for creating the *Z*-score map. Significance was defined as a *Z*-score above 2.81 (α < 0.025/10). The results of the ROI-based analyses are presented in Supplementary Table 3. Compared with those of the disease controls, in the present patient, gray matter volumes were significantly smaller in the left STG (*Z*-score = 4.28), left PT (*Z*-score = 3.88), and bilateral SMG (left: *Z*-score = 4.21; right: *Z*-score = 3.35).

##### ^123^I-Iodoamphetamine Single-Photon Emission Computed Tomography

To assess the patterns of hypoperfusion, ^123^I-IMP-SPECT data were analyzed with three-dimensional stereotactic surface projections using the normal perfusion database for a Siemens e-cam. There was hypoperfusion from the temporal to the parietal lobes of the left hemisphere, with a more intense reduction from the anterior temporal lobe to the middle and inferior temporal gyri (Figure 2C).

### Laboratory Examination

Beta-amyloid 1–42 (Aβ42) and phosphorylated tau protein (p-tau) in the patient's cerebrospinal fluid (CSF) were quantified on days 11 after admission. The patient's CSF Aβ42 level (1,023 pg/ml) was within the normal range, whereas the CSF p-tau level (68.3 pg/mL) was slightly elevated (reference value of <50 pg/ml) (30).

### Outpatient Follow-Up

At her last visit to the hospital (3 years after hospitalization scrutiny), she had developed marked aphasia with phonological mishearing and sentence comprehension disorder. She still understood the written word better than the spoken word, but her understanding of the written word was also greatly declined, due in part to her progressive semantic comprehension disorder. The effort of speech production was not increased, and prosodic articulation was intact. Although she could produce a meaningful speech, she talked about the same content with similar phrasing and only superficial. Behavioral disorders did not worsen, except for obsession with handwashing. She continued to do well physically, and her neurological examinations were unchanged.

## Discussion

Herein, we presented a case of svPPA with impaired verbal sound discrimination. Although spared repetition ability is one of the supported diagnostic features of typical svPPA (1), our patient sometimes made errors in repetition tasks. However, the errors tended to occur with short words rather than sentences, and she understood written words better than spoken words. These symptoms were not likely to be a repetition disorder because of impaired verbal short-term memory. Thus, we performed a detailed assessment of auditory functions, verbal sound discrimination/recognition, and neuroradiological analyses. The results revealed that her temporal auditory acuity and accuracy of phoneme recognition were decreased, and the gray matter volumes in the left STG, PT, and bilateral SMG were significantly smaller than that in our disease control patients with typical svPPA.

Several studies have reported that most of the patients with PPA complicated with impaired verbal sound recognition eventually demonstrate apraxia of speech, suggesting nfvPPA (6–8, 10–12, 31–37). In addition, some of them demonstrated behavioral disorders and were considered to have frontotemporal dementia (6–8, 10, 32, 38). Among the patients with PPA complicated with impaired verbal sound recognition, none had svPPA and only one lvPPA was reported (9), which was diagnosed as Alzheimer's disease by ^11^C-labeled Pittsburgh Compound-B [(11)C-PIB]-positron emission tomography. Thus, the presented case is the first case of svPPA with impaired verbal sound discrimination. Furthermore, the patient received sufficient clinical and neurophysiological assessments for auditory functions, which have been performed in limited studies. To confirm impairment of auditory verbal discrimination, systematic auditory examinations including auditory brainstem responses, pure-tone audiometry, and verbal sound discrimination/recognition are necessary. Only eight patients who were reported previously and our patient performed all these examinations (Supplementary Table 3) (6–9, 32, 34–36). In addition, temporal acuity, one of the causes of deficient verbal sound recognition (23, 24, 39), should be assessed by click counting and fusion tests.

There are two language processing pathways as follows: The dorsal (articulatory–phonological) pathway and the ventral (lexical–semantic) pathway (40). A recent model of the dorsal articulatory–phonological pathway proposes the existence of auditory–phonological representations of speech supported by the superior temporal cortex (41, 42). Johnson et al. (14) reported impaired auditory phonemic discrimination in lvPPA by assessing the discrimination of phonemes differing on a single acoustic characteristic and revealed that the impairment was positively correlated with the gray matter volume in the left angular gyrus through VBM on brain MRI. Hardy et al. (15) reported that patients with nfvPPA and svPPA have phonetic spectral processing deficit, which was “an analogous deficit has been demonstrated previously to affect a range of non-verbal sounds in both nfvPPA and svPPA,” and the deficit was associated with the gray matter volume in the left SMG. In addition, the case of lvPPA with pure word deafness (9) had cortical thinning in bilateral Heschl's gyri, PT, and superior temporal sulcus (STS), compared with healthy controls. The case of nfvPPA with auditory agnosia had hypoperfusion mainly in the left superior temporal and inferior frontal gyri (12). A recent study using activation likelihood estimation meta-analysis of 23 fMRI experiments identified significant activation likelihoods in the left mid-posterior STS with phonetic and phonological processes (43).

These studies have suggested the regions important for verbal sound discrimination are the STS, STG, and PT among the auditory-association cortices. In our patient, the results of the neuroradiological analyses using VBM and ROI-based analyses of MRI demonstrated markedly smaller gray matter volumes in the left STG, PT, and bilateral SMG than in those of typical svPPA. These findings indicated that dysfunction of these regions affects phonemic processes in the present case.

Our patient with svPPA exhibited impairment of verbal sound discrimination. We searched for case reports in PubMed, Web of Science, and Japanese academic journals. The search included the keywords “word deafness,” “auditory agnosia,” and “cortical deafness.” Then, we selected reports of patients with neurodegenerative diseases in which systematic auditory assessments, including pure-tone audiometry, verbal sound discrimination, and auditory brainstem responses, were performed. Eight patients fulfilled our criteria (Supplementary Table 3). Hypometabolic regions involved the temporal lobes: bilateral in five patients, left in two patients, and right in 1 patient. Impaired verbal or non-verbal sound discrimination may be associated with lateralized temporal lobe dysfunction. The degree of dorsal pathway impairment may affect the severity of verbal and non-verbal sound discrimination deficits.

The most common cause of svPPA is frontotemporal lobar degeneration associated with TDP43 type C pathology (44). However, recent studies (44–46) have reported other types of pathological changes, such as Alzheimer's disease (AD). Patients with svPPA and AD pathology demonstrated a more widespread cerebral hypoperfusion than patients with svPPA and TDP43 pathology. Hypoperfusion areas encompass the inferior parietal lobule, posterior cingulate cortex, and precuneus, similar to patients with typical AD (47–49). Brain atrophy extended to the inferior parietal lobule, and increased p-tau levels in the CSF suggested AD pathology in the present case.

This study had a few limitations. First, we could not match the age of the present patient and those of individuals in the control group with svPPA in the VBM and ROI-based analysis. We could not recruit age-matched disease controls because our patient was older than patients with typical svPPA. Second, we could not fully investigate the perceptive function of non-verbal sounds because many factors such as auditory apperceptive processing and semantic processing affected her performance (50–52). In addition, age-matched control data for non-verbal sound discrimination tasks were unavailable.

This case provides further evidence that the extent of lesions involved in each patient with PPA affects the types of hearing impairment. A comprehensive hearing assessment is important for patients with PPA at the early stage of the disease, which may predict the extent of cerebral dysfunctions and related pathology.

## Data Availability Statement

The original contributions presented in the study are included in the article/Supplementary Material, further inquiries can be directed to the corresponding author/s.

## Ethics Statement

Ethical review and approval was not required for the study on human participants in accordance with the Local Legislation and Institutional Requirements. The patients/participants provided their written informed consent to participate in this study. Written informed consent was obtained from the individual(s) for the publication of any potentially identifiable images or data included in this article.

## Author Contributions

NK acquired case data, designed the study and drafted the manuscript. AM, KK, YS, and EK acquired case data. NO designed some original tests. WN supervised the study. SK analyzed the data and supervised the study. KS supervised the study and helped to draft the manuscript. All authors contributed to the article and approved the submitted version.

## Funding

This work was supported by Health Labor Sciences Research [Grants Nos. 20GB1002 and 20GC1008], Grant-in-Aid for Transformative Research Areas [Grant No. 20H05956], Grant-in-Aid for Scientific Research (B) [Grant No. 21H03779] to KS, and Grant-in-Aid for Early-Career Scientists [Grant No. 19K20381] to NK.

## Conflict of Interest

The authors declare that the research was conducted in the absence of any commercial or financial relationships that could be construed as a potential conflict of interest.

## Publisher's Note

All claims expressed in this article are solely those of the authors and do not necessarily represent those of their affiliated organizations, or those of the publisher, the editors and the reviewers. Any product that may be evaluated in this article, or claim that may be made by its manufacturer, is not guaranteed or endorsed by the publisher.
