# Supplementary material for: Case Report: Semantic Variant Primary Progressive Aphasia With Impaired Verbal Word Discrimination
Source: Front Neurol. 2022 Jun 16;13:873735. doi: 10.3389/fneur.2022.873735 (PMC9243420; doi:10.3389/fneur.2022.873735)
Supplement: Supplementary file 1 [file Data_Sheet_1.pdf]

Supplementary Table 1. The results of non-verbal sound examination

		this patient	Disease control (mean±SD)
Recognition of environmental sounds accuracy		61% (22/36)	82.3 ± 6.1% (39.5 ± 2.9/48)
Recognition of Japanese nursery song (10)			
	Humming	10	10
	Pointing	4	10

Recognition of environmental sounds test

Forty-eight environmental sounds were presented binaurally in the following 8 categories: human nonverbal sounds created by the mouth and/or throat (e.g., cough), human sounds created by a body (e.g., handclap), an animal barking (e.g., dog), natural inanimate sounds (e.g., wind), sounds created by tools (e.g., scissors), sounds created by household appliances (e.g., cleaner), alarm sounds (e.g., car horns) and traffic sounds (e.g., airplane). Six pictures of the same category were presented at the same time, and the patient was asked to point to the picture that corresponded to the sound. As this patient could not recognize 12 target pictures, they were excluded from the analysis. In the remaining 36 trials.

Disease control is 6 patients with idiopathic normal pressure hydrocephalus (iNPH) (mean age 79 ± 3.8 years, 4 males and 2 females.) Although they were able to recognize all of 48 pictures, total number of pictures evaluated differed from this patient.

Recognition of Musical Tunes test

Instrumental melodies of 10 Japanese nursery songs were presented binaurally from a speaker. First, this patient was asked to sing or hum along with the songs. Although she was not able to recall the lyrics, she hummed melodies of all 10 songs. Second, we asked the patient to point to the picture, among 5 other pictures, corresponding to the lyric of each nursery song. She chose only 4 target pictures correctly. This test was only performed to two people. Patient 1 was 82 years old man without cognitive impairment and Patient 2 was 73 years old man with iNPH. They both answered all 10 nursery songs correctly.

Supplementary Table 2. Magnetic resonance imaging parameters.

Subjects	MRI unit	Sequence	Parameters
4 patients with typical SD (3 females and 1 male)	1.5-Tesla GE Signa (General Electric Company, Milwaukee, WI, USA).	3D-SPGR	Section: transverse<br>TR: 20 ms<br>TE: 4.1 ms<br>Thickness 1.5 mm<br>Number of slices: 108<br>Intersection gap: none FOV: 23 × 23 cm<br>Matrix: 256 × 256
The present patient<br>6 patients with typical SD (4 females and 2 males)	3-Tesla MAGNETOM Trio, A Tim System (Siemens AG, Medical Solutions, Erlangen, Germany)	3D-MPRAGE	Section: transverse<br>TR: 1900 ms<br>TE: 2.98 ms<br>Thickness 1.0 mm<br>Number of slices: 176<br>Intersection gap: none FOV: 21 × 21 cm<br>Matrix: 256 × 256

3D-SPGR: three-dimensional spoiled gradient echo; 3D-MPRAGE: three-dimensional magnetization-prepared rapid gradient-echo; FOV: field of view; SD: semantic dementia; TE: echo time; TR repetition time.

Supplementary Table 3. The results of regions of interest analyses

Regions	Hemisphere	Z-score	α
Heschel's gyrus	Left	-2.05	0.02018
	Right	-1.89	0.02938
Superior temporal gyrus	Left	4.28*	0.00001
	Right	2.14	0.01618
Planum temporale	Left	3.88*	0.00005
	Right	1.73	0.04182
Angular gyrus	Left	1.48	0.06944
	Right	2.69	0.00357
Supramarginal gyrus	Left	4.21*	0.00001
	Right	3.35*	0.00040

*Z >2.81 (α < 0.025/10)

Supplementary Table 4. Auditory, language and neuroimaging profiles of selected previous case reports and present case.

		Onset age	Sex	PPA subtype	Semantic impairment	ABR	PTA	PB-MAX	Environmental sound discrimination	Click count (/sec)	Click fusion (msec)	SPECT hypoperfusion or PET hypo-metabolism area
**1**	Otuki et al. (28)	60	M	Nonfluent	-	WNL	40-50	R: 0% (0/40 mora)<br>L: 0% (0/40 mora)	12/20 (p)	<3	R: 300<br>L: 200	Left temporal
**2**	Lee et al. (29)	67	F	Nonfluent	-	WNL	R: 61.3<br>L: 60.0	R: 0% (90 dB)<br>L: 0% (100 dB)	NR	NR	NR	WNL
**3**	Kaga et al. (30)	70	F	Nonfluent	-	WNL	Mildly elevated	R: 25%<br>L: 20%	0/24 (n)<br>4/24 (p)	NR	NR	Bil. auditory cortices, Broca's area, Wernicke's area, FTP (R>L)
**4**	Iizuka et al. (31)	64	M	Nonfluent	-	WNL	R: 22.5<br>L: 28.8	0% (30 dB)<br>5-75% (40-90 dB)<br>*presented to both ears	20/20	NR	NR	Bil. frontotemporal (R<L)
**5**	Kim et al. (32)	57	F	Logopenic	-	WNL	R: 35<br>L: 30	R: 20% (85 dB)<br>L: 32% (80 dB)	Animal 6/8<br>Music/song 4/6	NR	NR	Bil. temporoparietal areas (R<L)
**6**	Ota et al. (33)	68	F	Nonfluent	-	WNL	R: 26.3<br>L: 33.8	R: 80% (60dB)<br>L: 45% (45dB)	8/20 (n)<br>16/20 (p)<br>Amusia +	R: 3<br>L: 2	R: 87<br>L: 109	Bil. FTP area (R>L), right thalamus, basal ganglia
**7**	Satoh and Ito (34)	66	F	Nonfluent	-	WNL	WNL	35%	10/24<br>song 16/30	NR	NR	Right temporal
**8**	Sato (35)	60s	M	Nonfluent	-	WNL	R: 37<br>L: 35	50% (70 dB)	35/40<br>Amusia +	NR	NR	Left hemisphere, right parietal
**9**	Our Case	77	F	Semantic	+	WNL	R: 36<br>L: 36	R: 75% (65 dB)<br>L: 80% (65 dB)	29/48 (p)<br>Song (p): 4/11	R: 5<br>L: 5	R: 8<br>L: 8	Bil. ATL (R<L), left MTL, PTL, TPJ

Eight case reports were conducted systematic auditory examinations for judging auditory verbal discrimination impairment. Such as auditory brainstem responses, pure-tone audiometry and verbal sound discrimination and recognition. All of them were right-handed.

PPA = primary progressive aphasia, ABR = auditory brainstem response, PTA = pure-tone audiometry threshold (dB), PB-MAX = maximum recognition score in phonetically balanced word list , SPECT = single-photon emission computed tomography, PET = positron emission tomography, WNL = within normal limits, NR =not reported, R = right, L = left, mora = Japanese an ultimate

minimum unit of a verbal sound, (p) = pointing, (n) = naming, FTP = frontotemporoparietal area, ATL = anterior temporal lobe, MTL = middle temporal lobe, PTL = posterior temporal lobe, TPJ = temporoparietal junction
